# Exploring the lived experiences of women with multiple gestations in Iran: a phenomenological study

**DOI:** 10.1186/s12884-024-06384-4

**Published:** 2024-03-15

**Authors:** Zhina Banafshi, Alireza Khatony, Amir Jalali, Rostam Jalali

**Affiliations:** 1 Kermanshah University of Medical Sciences, Kermanshah, Iran; 2 Student Research Committee, Kermanshah University of Medical Sciences, Kermanshah, Iran

**Keywords:** Pregnancy, Multiple pregnancy, Women, Lived experiences, Phenomenological research, Qualitative research

## Abstract

**Background:**

Multiple gestations present numerous physical, psychological, social, and economic challenges for women. Understanding the problem-solving experiences of pregnant women carrying multiple can be invaluable. This study aimed to explore the experiences of Iranian women with multiple gestations.

**Methods:**

This descriptive phenomenological study utilized purposive sampling and continued until data saturation. Data collection involved in-depth semi-structured interviews, with analysis performed using Colaizzi’s 7-step method. MAXQDA software was employed for data management.

**Results:**

This study involved 12 women with multiple gestations. The average age of the participants was 33.76 ± 6.22 years, and 9 were pregnant with triplets. The data were categorized into four primary themes: the paradox of emotions, the pregnancy prison, immersion in fear, and the crystallization of maternal love, encompassing 17 sub-themes.

**Conclusion:**

Pregnant women with multiple gestations undergo various changes and experience conflicting emotions. Enhancing their ability to adapt to and accept numerous pregnancies can be achieved through supportive, personalized, and family-centered care, along with improvements and revisions in care policies for multiple gestations.

## Background

As a transformative life event, pregnancy ushers in a period filled with growth, hope, expectations, and concerns for women and their families [1]. It represents a transition into motherhood, necessitating a profound reevaluation of goals, roles, behaviors, and responsibilities as women cultivate a new self-image and embrace their maternal role [2]. However, this transition can be particularly complex for certain women, especially those with multiple gestations [3].

Multiple gestations refer to the simultaneous growth of more than one fetus in a mother’s uterus, resulting in the birth of numerous infants [4]. The global incidence of multiple gestations has surged by 70–75% [5]. In various regions of Iran, studies have reported twin prevalence’s ranging from 6.2 to 14 per thousand births, triplets from 0.15 to 0.7, and quadruplets at 0.09 per thousand births [6]. This substantial increase in multiple gestations can be attributed to the heightened use of ovulation-inducing agents [7], assisted reproductive techniques, older maternal age, and advancements in specialized prenatal and postnatal care [8].

Despite certain similarities between women’s experiences with singleton and multiple gestations, it is undeniable that the journey of numerous gestation and parenting differs significantly from that of singleton pregnancies [9, 10]. Multiple pregnancies carry inherent risks, such as intrauterine growth restriction (IUGR) [11], placental issues, elevated perinatal mortality rates, eclampsia, hormonal imbalances, blood disorders, and genitourinary complications [12]. Mothers with multiple gestations require more extensive medical and surgical interventions and are nearly six times more likely to require hospitalization due to pregnancy complications [13]. The uncertainty of the future, increased caregiving responsibilities, financial strains [14], and the gap between the support they need and what they receive further compound the challenges mothers face with multiple gestations [15]. They also contend with heightened fatigue, anxiety, and depression compared to mothers with singleton pregnancies [16], with significant depression affecting a third of mothers carrying twins [17].

The impact of multiple gestations extends beyond the physical and mental well-being of mothers and children, affecting various aspects of their social, economic, and familial lives. The rising prevalence of multiple gestations [9] underscores the urgency of addressing mothers’ concerns, needs, emotions, feelings, and perceptions in this unique situation. It should be noted that most of the existing studies investigating women’s experiences with multiple gestations have utilized a quantitative approach. Qualitative studies are necessary to obtain in-depth and rich data. Consequently, this study’s objective was to conduct a qualitative investigation to elucidate the lived experiences of Iranian women with multiple gestations. The results served as a valuable resource for nurses, clinical psychologists, counselors, and physicians better to understand the experience of Iranian women with multiple gestations and to implement appropriate interventions and approaches to support them effectively.

## Methods

### Research design

A descriptive phenomenological approach was employed to comprehend the lived experiences of mothers with multiple gestations, utilizing Colaizzi’s analysis method [18]. In this approach, the researcher explored the significance and essence of the phenomenon from the participants’ perspectives.

### Participants and sampling method

The participants consisted of women who had undergone multiple gestations in Kurdistan province, situated in the western region of Iran. The study was initiated with purposive sampling, and persisted until data saturation, meaning new information or insights were no longer emerging from the interviews. Inclusion criteria encompassed women with a history of multiple gestations, devoid of neurological and mental disorders, willing to share their experiences, and participating in the research voluntarily and consciously. By the 9th interview, no new themes or data emerged, and the data was saturated. To ensure this with more certainty, three additional interviews were conducted.

### Research tools

To collect data, comprehensive face-to-face semi-structured interviews were conducted. Alongside these discussions, detailed field notes were also recorded. The interviews began with foundational inquiries such as, “Could you delineate your experience with multiple gestations?” and “What is your comprehension of multiple gestations?” Subsequently, the researcher employed probing queries including “why,” “how,” and “please elaborate further” to elucidate the subject matter.

### Data collection method

Semi-structured, in-depth interviews were employed to gather data. The researcher visited health centers and maternity hospitals in Kurdistan province to select participants. Subsequently, interview locations and times were agreed upon with the participants. Twelve interviews were conducted, each lasting approximately 60 to 90 min. Eleven interviews were held at participants’ residences, while one occurred at Beasat Maternity Hospital in Sanandaj. Interviews were recorded with participants’ consent. The interviewer (the first author) was a Ph.D. student in nursing education and fluent in the participants’ native language (Kurdish). All researchers involved in this study were nursing professionals with expertise in qualitative research. Data Analysis followed the 7-step Colaizzi’s approach, conducted simultaneously with data collection [18].

Step 1: Repeatedly listening to recorded interviews and transcribing them verbatim, along with reviewing field notes, was done to gain a profound understanding of participants’ experiences.

Step 2: Multiple readings of the transcribed text led to the identification of 380 key terms.

Step 3: Summarize these key terms and explain their meanings in the participants’ language.

Step 4: Group the formulated meanings into thematic clusters; 170 definitions out of the initial 380 key terms were extracted and organized compared to the core terms. An experienced professor in qualitative studies oversaw this process.

Step 5: Develop comprehensive descriptions of the 170 identified meanings based on previously extracted definitions and organize them into 17 theme clusters and four main themes.

Step 6: Integrating the results to comprehensively describe the phenomenon by identifying relationships between the themes.

Step 7: Validating the primary structure of the phenomenon with the participants to ensure the results’ accuracy, clarity, and conciseness. MAXQDA software was utilized for data management.

### Trustworthiness

Four criteria - credibility, transferability, dependability, and confirmability - were applied, as per the guidelines of Lincoln and Guba, to ensure the rigor of the data [19]. After analyzing each interview, participants were recontacted to validate the content, enhancing credibility. Revision by both participants and external observers, coupled with ongoing communication and building trust with the participants, contributed to this credibility. Detailed explanations of each research step were provided to enhance dependability and facilitate accurate judgments during evaluation by others. Additionally, segments of interviews and their analyses were shared with other research team members who were experienced professors in qualitative research to bolster confirmability, thereby confirming the accuracy of the coding. Extensive efforts were made to fully elucidate the research context to enable readers to assess the transferability of the findings.

## Results

The study involved most participants who had experiences with triplet pregnancies (*n* = 9). The mean age of the participants was 33.76 ± 6.22 years, with the majority conceiving through assisted reproductive techniques (*n* = 9). Half of the participants had a familial history of multiple gestations; most were housewives (*n* = 10). Additionally, half of the participants (*n* = 6) were categorized as belonging to the middle economic status (Table 1).

**Table 1:** Demographic characteristics of the participants

Participant number	Age	Job	spouse’s job	Education	economic status	Number of Gestations	History of Abortion	Family History of Multiple Gestations	Type of Pregnancy (wanted or not)	Number of Children	Place of Interview	Place of Residence
1	35	Housewife	engineer	Master in Administration	middle	3	Yes (1)	No	Normal (wanted)	3	Participant’s house	Sanandaj
2	34	Housewife	Farmer	Under diploma	low	3	No	No	Use of ovulation induction drugs and fallopian tube blockage surgery(wanted)	3	Participant’s house	Isaabad Village
3	32	Employee	Employee	Master in environmental health	high	4	Yes (1)	Yes	Normal (wanted)	3	Participant’s house	Sanandaj
4	31	Housewife	engineer	Master in psychology	middle	3	No	No	Normal(unwanted)	3	Participant’s house	Sanandaj
5	48	Housewife	Car Mechanic	Under diploma	low	3	No	Yes	Normal (unwanted)	6	Participant’s house	Sanandaj
6	41	Housewife	laborer	Certificate and associate degree in accounting	low	3	Yes (2)	No	Use of ovulation induction drugs and IVF(Wanted)	2	Participant’s house	Sanandaj
7	31	Housewife	Construction worker	Diploma	low	3	No	Yes	Use of herbal drugs (Wanted)	4	Participant’s house	Kamyaran
8	33	Housewife	Teacher	Under diploma	middle	3	Yes (2)	No	Use of ovulation induction drugs (Wanted)	3	Participant’s house	Sanandaj
9	29	Teacher	Teacher	Master in primary education	middle	3	No	No	Use of ovulation induction drugs (Wanted)	3	Participant’s house	Sanandaj
10	27	Housewife	shopkeeper	Diploma and Associate degree in sewing design	middle	3	No	Yes	Use of ovulation induction drugs(Wanted)	3	Participant’s house	Sanandaj
11	23	Housewife	shopkeeper	Diploma	high	2	No	Yes	Use of ovulation induction drugs and Fallopian tube blockage surgery (Wanted)	2	Beasat Hospital in Sanandaj	Marivan
12	30	Housewife	Employee	Under diploma	Middle	4	No	Yes	Taking medication to regulate menstruation (Wanted)	4	Participant’s house	Sanandaj

Upon data analysis, this research identified four primary themes and 17 sub-themes (Table 2). These overarching themes included the paradox of emotions, pregnancy imprisonment, immersion in fear, and the crystallization of maternal love.

**Table 2:** Extracted sub-themes and themes related to the experiences of women with multiple gestations in this study

Main Theme	sub-Themes	Main Codes
Paradox of Emotions	Hope in the heart of darkness	- Doubt and hope in the fetus’s health - Doubt and hope in completing the pregnancy - Doubt and hope in the diagnosis of multiple gestations
	Contradiction of happiness and sadness	- Valuable difficulty - Contradiction of happiness and sadness - The paradox of emotions about being overweight - Multiple Gestations as an opportunity in the heart of a threat
The pregnancy Prison	Physical and behavioral restrictions	- Feeling of lack of energy and fatigue - Inability to manage life affairs - Absolute Rest - Reducing risky behaviors - Restrictions on food intake
	The feeling of being trapped	- Counting the days for childbirth - Feeling of being trapped - Mother’s invisible problems - Feeling of lack of control over pregnancy - Feeling of exhausted
	Social restrictions	- Isolation - Lower job and educational opportunities - Difficult access to treatment facilities - Unreasonable expectations
	Economic restrictions	- High costs of treatment and diagnostic procedures - Poor housing condition - Fear of inability to bear the costs of raising children - Inadequate insurance coverage
	Inadequate Health System Services	- Unnecessary tests and examinations - Care without compassion from the treatment staff - Unfavorable facilities and equipment - The unfamiliar and stressful hospital setting - Disclosing the news of multiples in an inappropriate way
Immersion in Fear	Overwhelming Shock	- Shock and neglect - Breaking the fertility rules - Being abandoned in the path of the unknown - Contrast of expectation and reality
	Inevitable changes	- Musculoskeletal pains - Skin and hair problems - Hormonal problems - Digestive problems - Disrupted sleep patterns - Genital and urinary problems - Cardiovascular problems - Placental problems - Multiple hospitalizations - Abnormalities in blood tests - High blood pressure - Physical and appearance changes - Changes in lifestyle
	Inner Turmoil	- Self-blame - Decreased self-image - Decreased self-confidence - Seeking induced abortion - Disrupting the routine of life - Feeling confused
	Psychological and emotional anxieties	- Fear of abortion - Fear of premature birth - Fear of the future - Fear of fetal abnormalities - Fear of maternal complications
	Stress-Exacerbating Factors	- History of abortion - History of fetal abnormalities - History of infertility - Negative attitude towards Multiple Gestations - Mother’s underlying disease - Unplanned pregnancy - Social network-induced stress - The medical staff-induced stress
The crystallization of Maternal Love	Fulfilling the dream of motherhood	- Enduring hardships for the love of children - Higher self-care - Mother’s devotion - The fetus’s health is the most crucial priority of the mother
	Heartwarming Connection with the Fetus	- Fetal movements that increase maternal love - Creation of a sense of belonging - The healing sound of the fetal heart - a pleasant connection with the fetus - Distinguishing a separate identity for the fetus
	A Sense of Personal Growth	- Pregnancy as the complementation of a woman’s identity - Pregnancy as the complementation of the whole family - Changing the way of looking at life - Pregnancy as an opportunity for growth and excellence
	Peace of mind	- Consolatory support from husband and family - Connecting to supernatural power - Multiple Gestations as the fulfillment of a dream - Multiple Gestations as a compensator for years of infertility
	Feeling happy	- Pregnancy as a marital lifesaver - Happiness from the fetus’s growth - Beautiful waiting - Feeling proud

## Paradox of emotions

### Hope in the heart of darkness

Despite the unique challenges and stresses associated with multiple gestations, pregnant women often maintain a sense of hope amidst despair. A 31-year-old housewife and mother of triplets shared her perspective:

*“Multiple gestation is an enigmatic journey. Just when everything seems to be going well, something unexpected occurs, throwing everything into disarray. However, I always clung to hope. For instance, I was deeply concerned when the sonographer informed me that one of my children had a large neck and recommended an amniocentesis. Despite my worries, deep down, I remained convinced that my children were healthy.”*

Another 31-year-old housewife, holding a master’s degree in psychology and also a mother of triplets, shared her perspective:

*“Despite being very sick during my pregnancy, my heart weakened, and the doctors mentioned the possibility of miscarriage. However, I remained hopeful that these difficult days would come to an end and that I would be able to embrace my children with love. I endured all these hardships with the hope of holding my babies in my arms.”*

### Contradiction of happiness and sadness

Participants frequently grappled with the contradiction between their desire for pregnancy and childbearing on the one hand and the fear and stress associated with multiple gestations on the other. This led to simultaneous experiences of mixed emotions, such as sadness, joy, opportunity, and threat during pregnancy. A 35-year-old housewife and mother of triplets expressed her sentiments:

*“Triplet pregnancy is like a two-sided coin. When I discovered I was pregnant with triplets, I did not know whether to cry or laugh. On the one hand, I was elated because I would have three children in a single pregnancy. Still, on the other hand, I was despondent due to the complexity and difficulty of a triplet pregnancy. I was apprehensive about the challenges of raising three children.”*

A 29-year-old employed mother of triplets reflected on her journey:

*“Pregnancy with multiple babies was like a sweet dream for me, but when the pregnancy complications started, and one of the fetuses encountered a problem, the sweetness of pregnancy gradually turned bitter for me. Sometimes, I didn’t know whether to be happy or upset about having triplets. “*

## The pregnancy prison

### Physical and behavioral restrictions

Women with multiple gestations face a plethora of physical, behavioral, and dietary constraints, necessitating adjustments to their activities and lifestyles to accommodate their pregnancies. A 30-year-old housewife and mother of quadruplets articulated her experience, stating:

*“I used to be quite fearless before conceiving, with no reservations. However, my approach shifted significantly upon becoming pregnant. I’ve become exceedingly cautious, particularly concerning my children’s well-being. I’ve been hyper-vigilant, avoiding stress, certain foods, and limiting my outings.”*

Similarly, a 32-year-old working mother of quadruplets recounted her ordeal:

*During the latter stages of my pregnancy, I underwent cervical cerclage and was confined to complete bed rest. Simple tasks such as using the bathroom became insurmountable challenges, necessitating a bedpan. I experienced significant incapacitation and felt deeply embarrassed by my predicament.*

### Feeling of being trapped

Multiple gestation imposes constraints on women, often making them feel their freedom has been stripped away, confining them within the confines of pregnancy. A 31-year-old housewife and mother of triplets shared her experience:

*“During the last few months, I was confined to complete rest. I was home alone from morning till night, feeling like a prison. Every day, I yearned for a glimpse of the world outside. I was isolated at home, and time seemed to stand still. Each day was monotonous, and I was overwhelmed by feelings of depression. I longed to give birth and move past this period with each passing day.”*

A 27-year-old housewife who is a mother of triplets shared her emotional struggle:

*After we were diagnosed with multiple gestations, my husband insisted that I should no longer work and even forbade me from leaving the house alone. He used to say, “I spent much money to get you pregnant; I don’t want to lose any embryos.” No matter what I did, he found fault with me, as if I were imprisoned during my pregnancy while he played the role of a prison guard.*

### Social restrictions

Multiple gestation imposes significant limitations on women’s career and educational pursuits, imposing notable social constraints on their participation. A 32-year-old working mother of quadruplets recounted her ordeal:

*“When my colleagues discovered my quadruplet pregnancy, they swiftly stripped me of my supervisory role, effectively demoting me. Any aspirations I voiced were promptly dismissed, with my multiple pregnancies cited as a hindrance. Even my husband advised me to resign from my job upon learning about the quadruplets.”*

Similarly, a 31-year-old homemaker holding a master’s degree in psychology and mother of triplets shared her experience:

*“Prior to discovering my pregnancy, I had undertaken a university exam. Initially, I didn’t foresee any issues as many individuals manage to study alongside caring for their infants. However, upon learning about my triplets, I promptly withdrew from university; the prospect of studying with three newborns seemed insurmountable.”*

### Economic restrictions

Managing multiple gestations imposes significant financial burdens on women and their families, including healthcare expenses and acquiring baby essentials. A 41-year-old housewife, the mother to triplets, described her situation:

*“My husband is employed as a laborer, and I have depleted our savings on IVF treatments, pills, and injections. With my husband lacking stable employment and health insurance, the costs associated with ultrasounds alone tripled compared to singleton pregnancies. Moreover, the expenses related to prenatal visits and medications proved overwhelming. We experienced immense pressure and were compelled to forgo certain necessities, including screenings.”*

A 27-year-old housewife, also a mother of triplets, expressed her emotional struggle:

*“Managing a triplet pregnancy differs significantly from a singleton pregnancy, requiring numerous doctor visits, tests, and ultrasounds, all of which come with considerable expenses. Financially, it has placed a significant strain on both my spouse and me. To afford the amniocentesis procedure, I even had to sell my wedding ring.”*

### Inadequate health system services

Throughout their pregnancies, women reported inadequate and unsympathetic support and care from medical personnel, often enduring both their behavior and the distressing hospital environment. A 48-year-old housewife, the mother of triplets, expressed her ordeal:

*“The hospital environment was exceedingly disheartening and affected our mental well-being. Physicians and nurses treated me disrespectfully, almost as if my triplet pregnancy was inconvenient. They seldom paid heed to my concerns, responding with indifference and bitterness. I felt compelled to endure their mistreatment as there was no room for complaint.”*

The 34-year-old mother of triplets in the village discussed her challenges with accessing medical services:

*Unfortunately, our village lacks a health center, so I had to visit the health center in a neighboring village, which also had limited facilities. For routine check-ups and tests, I had to travel to the city. When winter arrived, the roads became completely impassable. I felt extremely unwell and experienced vaginal bleeding. You cannot fathom the unfortunate circumstances I faced to reach the hospital. My children and I were about to die*.

## Immersion in fear

### Overwhelming shock

Most participants expressed profound shock upon learning about their multiple gestations, characterizing it as an unexpected turn of events. A 31-year-old housewife who is a mother of triplets shared her experience:

*“When I went for an ultrasound, and the doctor informed me that I was pregnant with triplets, I could not fathom that such a thing had occurred to me. I initially thought she was jesting. The shock was so immense that I sought another ultrasound to confirm the news.”*

### Inevitable changes

Women undergoing multiple gestations often experience numerous physical changes and frequently encounter complications, including skeletal pain, digestive issues, gestational diabetes, and preeclampsia. A 29-year-old employed mother of triplets recounted her challenges:

*“I encountered numerous physical hardships during my pregnancy. In the early months, I battled severe nausea and vomiting, which led to multiple hospitalizations. By the 25th week, I had developed gestational diabetes. Toward the end of the sixth month, I experienced severe headaches, elevated blood pressure, proteinuria, and kidney dysfunction. The doctors diagnosed it as pregnancy-induced hypertension and recommended an earlier cesarean section.”*

A 35-year-old woman, a housewife and a mother of triplets shared her thoughts and feelings.

*I truly paid a price for this pregnancy. I lost my beauty, attractive figure, and long hair forever. This pregnancy aged me by 10 years. During the pregnancy, I gained 22 kg, and my skin became covered in stretch marks. At a young age, I developed lower back pain. My belly was so prominent that sometimes breathing was difficult for me, and at night, I couldn’t sleep due to shortness of breath.*

### Psychological and emotional anxieties

Multiple gestation pregnancies frequently evoke significant psychological and emotional concerns for women. Worries regarding abortion, fetal abnormalities, and maternal complications are common anxieties among expectant mothers. A 23-year-old housewife, who is a mother of twins, articulated her concerns:

*“I constantly fretted about potential issues affecting both my babies and myself. The persistent stress often led to nighttime nightmares, where I feared developmental delays or miscarriages.”*

Similarly, a 35-year-old housewife and mother of triplets conveyed her sentiments:

*“I was extremely anxious about the prospect of terminating the pregnancies once again, knowing that this was my final opportunity to become a mother. If I chose to terminate the pregnancy, I would undoubtedly lose my husband. An unemployed, infertile widow lacks support and faces a grim future. Consequently, I was terrified of undergoing an abortion.”*

### Inner turmoil

Many pregnant women grappling with multiple gestations contend with inner turmoil, disrupted self-perception, diminished self-esteem, and feelings of powerlessness. Self-blame and persistent ruminations are common. A 27-year-old housewife who is a mother of triplets shared her emotional struggle:

*“I found myself in a state of confusion, unsure how to navigate these challenges. My control over my body had waned, and I harbored self-loathing. The weight gain of 25 kilograms left me feeling disfigured and unattractive, so I could not bear to look at myself in the mirror.”*

A 41-year-old housewife, also the mother of triplets, shared her emotional struggle:

*When I found out that one of my children had miscarried due to uterine growth restrictions, I was consumed by guilt and self-blame. I felt like I had interfered with God’s plan by resorting to IVF and forcing myself to become pregnant. I questioned whether I deserved to be a mother, especially since one of them was already taken from me. Likewise, I was stressed and didn’t know how to prevent losing the other two.*

### Stress-exacerbating factors

Numerous factors exacerbate the fear and stress experienced by mothers with multiple gestations. These factors include a history of abortion, fetal abnormalities, a negative perception of multiple gestation, and underlying women’s health issues. A 32-year-old employed mother of quadruplets shared her perspective:

*“I had a previous twin pregnancy that I had to terminate due to fetal abnormalities, which only heightened my stress. Moreover, the influence of social networks and medical professionals added to my anxiety and apprehension.”*

## Crystallization of maternal love

### Fulfilling the dream of motherhood

Most mothers carrying multiple gestations invest significant effort, energy, and resources to ensure the survival and growth of their fetuses. They prioritize their babies’ well-being over their own. A 31-year-old housewife who is a mother of triplets expressed her commitment:

*“I felt profound fear when I discovered I was carrying multiples and realized that my heart medications could harm my babies. My children’s health became paramount to me, surpassing my own, which led me to discontinue my medications in pursuit of healthier babies.”*

A 29-year-old working mother of triplets shared her dedication:

*“I am willing to do whatever it takes to ensure a healthy birth for my children. I have diligently followed all the instructions given by the doctors for eight months. Similarly, I have refrained from wearing makeup, avoided consuming outside food, and abstained from visiting others’ houses, fearing that I might contract an illness. Despite experiencing severe migraines, I refrained from taking any painkillers, fearing potential harm to the baby.”*

### Heartwarming connection with the fetus

The connection mothers form with their fetuses is a heartwarming and profoundly gratifying aspect of pregnancy. This bond becomes even more vital when they hear their babies’ first heartbeats and feel their movements. A 33-year-old housewife who is a mother of triplets shared her emotional experience:

*“The moment I heard the heartbeat of my babies, all my worries melted away. The sound of their hearts brought me immeasurable joy. Whenever I felt their movements in my womb, tears of happiness welled up. It was a profound and tender maternal sensation.”*

A 23-year-old woman, a housewife and mother of twins, expressed her emotions:

*Experiencing a twin pregnancy is a truly unique and exhilarating journey. It feels incredible to sense their playful movements inside my belly, as if they are kicking and interacting with me. Witnessing their growth and feeling their movements from within is a beautiful sensation. They truly understand my emotions, as their movements would intensify when I was happy and calm down when I was upset.*

### A sense of personal growth

Many participants consider multiple gestations as an opportunity for personal growth and gaining valuable experiences in embracing their roles as mothers. A 29-year-old employed mother of triplets reflected on her journey:

*“Managing multiple gestations was undeniably challenging, but I believe those hardships equipped me to be a better mother to my children. During pregnancy, I underwent significant personal growth, adopted a new perspective on life, and learned to cultivate patience and responsibility.”*

*A 34-year-old mother of triplets reflected on her journey*:

*“I used to feel a lot of humiliation and inadequacy because I couldn’t conceive before. However, now that I’m pregnant with triplets, I take pride in myself and feel a sense of fulfillment that I could complete my family and exceed expectations in fulfilling my role as a wife to my husband. Now I feel whole as a woman.”*

### Peace of mind

Most participants cited their belief in a higher power, their families’ unwavering support, and especially their husbands’ presence as sources of solace and peace of mind. A 41-year-old housewife, who is a mother of triplets, shared her sentiments:

*“After enduring a decade of infertility, my triplet pregnancy felt like a sweet dream that transformed my life. For the first time, I had my husband and family by my side, providing unwavering support. I experienced a sense of peace that had eluded me for years.”*

### Feeling happy

Some participants expressed immense pride and happiness regarding their multiple gestation pregnancies, considering them a source of joy and a divine gift. A 34-year-old mother of triplets conveyed her emotions:

*“I am thrilled that I conceived triplets. It fills me with pride and happiness. God bestowed His grace and mercy upon me, especially when my husband contemplated divorce due to our infertility struggles. This was undoubtedly the most significant event in my life.”*

A 33-year-old housewife who is a mother of triplets shared her emotional experience:

*“Ever since I discovered that I will have triplets, the world has become more vibrant. Everything seems more beautiful now. I used to long for just one child, but now I will have three. I have never been this happy before.”*

## Discussion

The present study aimed to explore women’s comprehension of multiple gestations. Each participant harbored distinct interpretations of multiple gestations rooted in their mental imagery and self-perception, influenced by various factors, including economic status, support systems, history of infertility, and personal attitudes. The participants viewed multiple gestations as a substantial challenge affecting every facet of their personal, social, and economic lives. Four primary themes emerged from the participants’ interpretations and statements regarding the significance of multiple gestations: The paradox of emotions, the pregnancy prison, immersion in fear, and the crystallization of maternal love.

The paradox of emotions was a noteworthy finding in the present study, encompassing themes of hope amidst darkness and the contradiction of happiness and sadness. Previous studies have shown that pregnant women with multiple gestations experience many emotions, ranging from elation and peace of mind to shock and anxiety; these emotional conflicts can intensify with an increase in the number of fetuses [20, 21]. The present study’s findings align with previous research examining the psychological manifestations of multiple pregnancies. Given that most of the participants in the present study experienced triplet pregnancies or more, they grappled with this emotional paradox. Some women regarded multiple gestations as a positive event and a divine gift, yet they felt discomfort and uncertainty due to the lack of preparedness and the challenges of pregnancy, resulting in a simultaneous mix of sadness and happiness. Furthermore, many participants had previously experienced periods of infertility, leading them to view multiple gestations as a golden opportunity to have multiple children. However, due to its high risks, they also perceived multiple gestations threatening their motherhood dream.

The pregnancy prison was another significant outcome of the present study, characterized by physical and behavioral restrictions, the feeling of being trapped, social limitations, economic restrictions, and inadequate healthcare services. In line with our study, previous research has also indicated that multiple gestation and fulfilling the maternal role can jeopardize women’s freedom, goals, and plans, imposing numerous physical and social restrictions; these restrictions become even more pronounced in twin pregnancies and beyond [22, 23]. They may even jeopardize women’s employment status, resulting in significant economic constraints [24]. Additionally, due to the heightened healthcare needs of women with multiple pregnancies, they encounter various health-related restrictions [25]. Our findings corroborate these studies and underscore the need for easy and affordable access to healthcare services for pregnant women with multiple gestations. Therefore, healthcare must address this issue. Generally, these restrictions associated with multiple gestations make women feel trapped in pregnancy confines. Given the increased sensitivity and responsibility associated with multiple gestations and mothers’ heightened responsibility for fetal health, women tend to exercise greater self-control over their behaviors. They believe their actions directly impact fetal health, motivating them to lead a healthier lifestyle. Moreover, societal and familial expectations within the patriarchal and traditional context of the Kurdish culture exacerbate these restrictions, as women are considered solely responsible for infant health.

Consequently, society expects women to dedicate their lives to pregnancy and child-rearing. Therefore, the perception of increased responsibility associated with multiple gestations compared to singleton pregnancy marks the inception of extensive social restrictions, hindering women from pursuing their goals, careers, and academic aspirations. Furthermore, multiple gestation imposes significant economic constraints on women, as most of the participants in the present study had undergone expensive infertility treatments and required additional health and medical services during pregnancy. The lack of adequate insurance coverage placed immense financial strain on these women.

Immersion in fear was a critical finding of the present study, encompassing themes such as overwhelming shock, inevitable changes, inner turmoil, stress-exacerbating factors, and psychological and emotional anxieties. These findings align with previous research indicating that multiple gestation is a complex biological-psychosocial phenomenon, leading to numerous physical changes [26], adverse maternal and fetal complications, and consequences related to multiple gestations, such as abortion, restricted fetal growth, placental problems, diabetes, and pregnancy-induced hypertension [11, 12, 27]. In addition to physical challenges, pregnant women with multiple gestations experience heightened levels of fear and anxiety [17, 20, 28]. In line with this study, Mikhailov et al. (2021) also showed that multiple pregnancies are an unexpected and critical event for women that disrupts their family regulation and causes psychological turmoil [29]. Also, previous studies have shown that several factors, including infertility experiences, challenging pregnancy records, and false information in social networks, can intensify the stress of women pregnant with multiple gestation [30, 31]. In our study, most participants had experienced infertility and lacked a family history of multiple gestation, making twin pregnancies or more an unusual and abstract concept that left them feeling shocked, confused, and fearful.

Furthermore, they grappled with profound psychological and emotional fears, including the fear of abortion, fetal abnormalities, postpartum complications, and lifelong repercussions of pregnancy, due to their understanding of the potential risks associated with multiple gestation, physical changes, and complications endured during their pregnancy. These fears were more pronounced in women with a history of abortion, extended periods of infertility, and underlying medical conditions, leading to increased feelings of insecurity. As women perceived more significant risks to their health and the health of their fetuses, their experiences of fear and anxiety intensified.

The crystallization of maternal love emerged as a significant discovery in the current study, delineated by thematic clusters encompassing the fulfillment of the dream of motherhood, heartwarming connection with the fetus, attaining peace of mind, feeling happy, and experiencing a sense of personal growth and happiness. These experiences are consistent with the findings of a study conducted by Ranjbar et al., which reported that mothers carrying multiple gestations labor tirelessly to achieve their ultimate goal of giving birth to healthy fetuses; they demonstrate remarkable resilience, often disregarding the challenges inherent in multiple gestations, driven by sacrifice and selflessness [32]. The love of a mother serves to sweeten the burdens and tribulations of pregnancy in such cases, with women proudly embracing their multiple gestations as a catalyst for personal development [33] and as an opportunity to fortify the foundation of their families [20]. Given that many participants in this study had a history of infertility, multiple gestation represented a miraculous blessing and divine reward. It allowed these women to compensate for years of infertility, ultimately fulfilling their identity as women. Within Iran’s traditional society and culture, bearing a child was essential to a woman’s identity. Multiple gestations presented an avenue for developing and affirming a woman’s identity [32], strengthening marital bonds, fostering trust, and elevating a woman’s social standing within the family structure. Women found themselves empowered, productive, and profoundly proud of carrying several embryos, signifying the culmination of their childbearing journey. This deep-seated joy intensified with the sensation of the fetus’s initial movements and the profound realization of motherhood. The emotional connection between mother and fetus proved instrumental in navigating the challenges associated with multiple gestations and facilitating maternal adaptation.

### Strengths and limitations

One of this study’s strengths lies in its participants’ diversity. Women with multiple gestations from urban and rural areas, encompassing varying levels of education, occupation, and economic status, were included. This broad spectrum of participants offers a comprehensive and profound insight into the study’s findings. Conducted among women with multiple gestations in Kurdistan, who are part of the Kurdish ethnic minority in Iran, the study underscores the necessity of exercising caution when extrapolating the results to other populations. It’s important to note that there could be a temporal discrepancy between the pregnancy and the interview, potentially impacting the accuracy of participant recollections. Over time, specific details about the pregnancy may fade from memory or become altered.

## Conclusion

Multiple gestation poses significant challenges across various aspects of women’s lives. Women carrying multiple fetuses face numerous changes, physical issues, and social, economic, physical, and behavioral constraints. Moreover, they contend with various conflicting emotions, including fear, apprehension, astonishment, happiness, and personal growth. Economic circumstances play a crucial role in influencing women’s emotions and experiences during multiple gestation, impacting the health status of both the mother and the fetuses, the available support system, the emotional bond between mother and fetus, the type of fertility treatment received, past pregnancy experiences, and a history of infertility. These experiences can have profound effects on women’s well-being, fetuses, and families. Understanding the challenges faced by pregnant women dealing with multiple gestation provides valuable insights for healthcare providers and policymakers in the field of women’s health. It helps identify these women’s psychological, social, physical, and economic hurdles. Consequently, policymakers can support the journey of multiple gestations by providing comprehensive and sustained support, optimal facilities and resources, and legislation to safeguard women’s rights and well-being.

## Data Availability

Datasets are available through the corresponding author upon reasonable request.

## Declarations

### Standard of reporting

COREQ guidelines were followed.
